# Clinical Trajectory of Late‐Onset Delusional Disorder Toward Evolving Dementia With Lewy Bodies

**DOI:** 10.1111/psyg.70236

**Published:** 2026-11-04

**Authors:** Hiroshige Fujishiro, Kazuhiro Takeda, Kuniyuki Iwata‐Endo, Shusei Arafuka, Seiko Miyata, Kunihiro Iwamoto, Youta Torii, Shuji Iritani

**Affiliations:** ^1^ Department of Psychiatry Nagoya University Graduate School of Medicine Nagoya Aichi Japan; ^2^ Department of Psychiatry Okehazama Hospital Fujita Mental Care Center Toyoake Aichi Japan

**Keywords:** Alzheimer disease, Lewy body disease, mild behavioural impairment, Parkinson's disease, pre‐psychosis, REM sleep behaviour disorder, schizophrenia

## Abstract

Recent clinical observations indicate that late‐onset psychosis can precede the onset of dementia with Lewy bodies (DLB). However, its longitudinal clinical trajectory remains poorly understood. Delusional disorder (DD) in older adults is relatively uncommon and poor insight frequently leads to refusal of psychiatric care, limiting available data despite its long‐standing diagnostic recognition. This article focuses on late‐onset DD in relation to subsequent development of DLB. We revisited our previously reported cases in which delusions were the initial manifestation of Lewy body disease (LBD) and aimed to identify the shared clinical features to help delineate more homogeneous subgroups within the heterogeneous spectrum of late‐onset psychosis. These cases with late‐onset DD revealed a recurring phenotype characterised by onset in the 50s and 60s, reluctance to seek psychiatric care, eventual hospitalisation, poor antipsychotic tolerance and delayed emergence of dementia accompanied by core features of DLB. Clinicopathological examination of four cases raises the hypothesis that persistent delusions may emerge in the presence of relatively restricted Lewy pathology, preceding the development of widespread cortical involvement and overt dementia. Relative preservation of neuronal populations in the substantia nigra may account for the absence of parkinsonism during the early phases dominated by isolated delusions. Despite long‐standing delusions beginning in the 50s and 60s, a common age range for DLB onset (mid‐70s and older) was observed, indicating an exceptionally prolonged prodromal period. These findings suggest that late‐onset DD associated with LBD may follow a clinical trajectory distinct from that of mild cognitive impairment with Lewy bodies (MCI‐LB), the most common clinical phenotype of prodromal DLB. Differentiating DD from broader late‐onset psychosis categories may facilitate future research by reducing heterogeneity and stratifying cases with potentially prolonged periods without dementia or parkinsonism. Clinical and pathological reports of late‐onset DD remain scarce and further case documentation is warranted.

## 1 Introduction

Late‐onset psychosis remains an insufficiently defined and conceptually unsettled field in psychiatry [1]. According to the *Diagnostic and Statistical Manual of Mental Disorders, Fifth Edition*, Text Revision (DSM‐5‐TR) criteria, schizophrenia is clinically characterised by hallucinations, delusions, disorganised thoughts and behaviour and negative symptoms, with a typical onset in late adolescence or early adulthood [2]. In contrast, delusional disorder (DD) is defined as the presence of one or more delusion(s) lasting ≥ 1 month(s), in the absence of the other manifestations characteristic of schizophrenia [2]. Although DD can occur in younger age groups, the condition is more prevalent in older individuals.

As the underlying pathophysiology of late‐onset psychosis remains unclear, the International Late‐Onset Schizophrenia Group proposed an influential age‐based classification system in 2000. The consensus classifies onset after 40 years of age as late‐onset schizophrenia (LOS) and onset after 60 years of age as very LOS‐like psychosis (VLOSLP) [3]. Both LOS and VLOSLP are defined as the emergence of hallucinations or delusions in the absence of mood disorders, without overt focal or progressive structural brain diseases at presentation. Therefore, these categories encompass a heterogeneous group of diagnoses, including schizophrenia and DD according to DSM‐5‐TR, rather than a single nosological entity. Although dementia is excluded by definition at the time of VLOSLP diagnosis, a recent systematic review reported a substantially increased risk for subsequent dementia in patients with VLOSLP compared with age‐matched controls [4]. In a Swedish population‐based cohort study, 17.8% of 15 409 patients diagnosed with VLOSLP subsequently received a diagnosis of dementia during follow‐up, compared with 7.1% of 154 090 matched controls (adjusted hazard ratio, 4.22). Among patients who developed dementia, the median interval from VLOSLP diagnosis to dementia diagnosis was 1.9 years, compared with 9.1 years in controls [5]. However, VLOSLP encompasses heterogeneous non‐affective psychotic disorders, including DD and the risk of dementia specifically associated with late‐onset DD remains unclear. Moreover, biomarker studies have reported higher positivity rates for Alzheimer's disease (AD) and dementia with Lewy bodies (DLB)‐related biomarkers among individuals initially diagnosed with VLOSLP [6, 7, 8, 9, 10]. These findings raise the possibility that a clinically meaningful subset of late‐onset psychosis represents a prodromal stage of a neurodegenerative disease rather than a primary psychiatric disorder. In clinical practice and research settings, these conditions are examined within a single framework without sufficient differentiation, resulting in heterogeneous clinical profiles, courses and potentially underlying mechanisms being considered together.

The recent progress in disease‐modifying therapies has further highlighted the importance of accurate etiological diagnoses at the prodromal stage of dementia. Within this context, DLB has attracted particular attention due to its long prodromal phase and well‐documented association with psychiatric symptoms, including hallucinations, delusions, depression and anxiety [10, 11]. Isolated rapid eye movement (REM) sleep behaviour disorder (RBD), a recognised prodromal manifestation of synucleinopathies, is frequently accompanied by psychiatric diagnoses years or decades before the onset of dementia [12, 13, 14].

In 2020, the research criteria for prodromal DLB formally recognised three clinical subtypes: mild cognitive impairment with Lewy bodies (MCI‐LB), delirium‐onset DLB and psychiatric‐onset DLB [10]. Among these, psychiatric‐onset DLB is characterised by late‐onset psychiatric symptoms, most commonly depression and psychosis, which precede the onset of cognitive decline and parkinsonism. These presentations are sometimes severe and require hospitalisation. Previous clinicopathological studies have reported that patients with autopsy‐confirmed Lewy body disease (LBD) exhibit clinical presentations consistent with late‐onset psychosis; however, these cases have been documented under a broad range of diagnostic labels, such as senile psychosis and a unified description of the condition remains lacking [15, 16, 17, 18, 19]. For instance, Nagao et al. demonstrated that 6 of 23 patients with LOS and DD (mean age at onset, 63.3 ± 12.9 years) were pathologically confirmed to have LBD, but they did not specifically investigate late‐onset DD as a distinct entity clearly separated from LOS [19]. Consequently, the relationship between psychiatric‐onset DLB and established psychiatric diagnoses like DD remains insufficiently defined [11].

In this article, we focus on late‐onset DD and present an integrated review of the literature with our case reports addressing DD associated with LBD [20, 21, 22]. Aligning cases of LBD according to shared underlying pathology and clinical course and extracting common clinical features may help delineate more homogeneous subgroups from the heterogeneous population of late‐onset psychosis. We first reviewed articles regarding DD in late life and compared it with the recent concept of pre‐psychosis, positioned within the framework of mild behavioural impairment (MBI). We subsequently revisited our previously reported cases in which late‐onset DD was associated with LBD. This review aimed to clarify the current understanding of DD in late life, characterise psychiatric‐onset LBD manifesting as DD based on our published cases, discuss the neuropathological substrates of persistent delusions preceding the onset of DLB and consider isolated delusions within the context of the long‐term course of LBD.

## 2 DD in Late Life

### 2.1 Prevalence of DD in Late Life

Although late‐life DD is encountered in routine clinical practice, its pathological mechanisms are yet to be fully elucidated and epidemiological data regarding DD are limited. The lifetime prevalence of DD is estimated to be approximately 0.2% [2]. Copeland et al. [23] reported a prevalence of 0.04% in a registry study of 5222 individuals ≥ 65 years of age.

The onset of DD typically occurs in middle or late adulthood [24]. A case register study examined the 1‐year prevalence of DD based on the DSM‐IV‐TR among individuals aged 60 years and older in a psychiatric catchment area in Amsterdam, the Netherlands [25] and determined the overall one‐year prevalence of DD to be 0.03%. DD was found only in women aged 70 years and older. According to the DSM‐5‐TR, DD can occur at a younger age; however, this condition may be more prevalent among older individuals. A recent Australian study by Nagendra and Snowdon [26] examined all patients with DD referred to a public geriatric psychiatric service over a 12‐year period. Within a large catchment area, only 55 individuals ≥ 65 years of age fulfilled the strict diagnostic criteria for DD, 39 of them female (70.9%), highlighting the challenges of case ascertainment rather than the true rarity of the disorder. The mean (± standard deviation [SD]) age at presentation was 74.5 ± 7.65 years (range, 65–101 years) and the mean age at onset was 67.5 ± 12.44 years (range, 30–98 years).

### 2.2 Clinical Profiles of DD in Late Life

In the DSM‐5‐TR, DD is defined as the presence of ≥ 1 delusion(s) lasting ≥ 1 month(s) in the absence of prominent hallucinations, disorganised speech, grossly disorganised or catatonic behaviour or negative symptoms characteristic of schizophrenia [2]. Psychosocial functioning is relatively preserved, apart from the impact of delusional beliefs and behaviour is not markedly bizarre or odd. The DSM‐5‐TR further classifies DD into subtypes based on the predominant delusional theme, including the persecutory, jealous, erotomanic, grandiose, somatic and mixed or unspecified types. Persecutory delusions have consistently been reported to be the most common presentation across all age groups. Among 55 patients with late‐life DD [26], the distribution of delusional subtypes was as follows: persecutory (87%), jealous (11%) and somatic (2%). Predominant persecutory delusions commonly involve beliefs of being poisoned, harassed or conspired upon by neighbours, caregivers or institutions. This finding has several important clinical implications. Persecutory delusions are frequently associated with interpersonal conflicts, mistrust of medical professionals, treatment refusal and delayed engagement with mental health services. Seventeen of the 55 patients (30.9%) refused medication or psychiatric care. During the observation period, 41 (74.5%) patients required inpatient treatment. Treatment outcomes were modest and complete remission was uncommon; however, atypical antipsychotics led to partial improvement in some patients. Consequently, many individuals with late‐life DD receive psychiatric attention only after prolonged periods of untreated illness, often precipitated by crisis events such as hospitalisation, legal involvement or threats to personal safety. Given the relative rarity and clinical characteristics of DD, data regarding this disorder in older adults remain limited despite its long‐standing inclusion in diagnostic classifications [27, 28]. Harris et al. [27] compared the profiles of cognitive deficits between 19 patients with late‐onset DD and 20 patients with AD in a memory clinic. According to DSM‐IV‐TR criteria for DD with an onset age of more than 65 years, the mean age and Mini‐Mental State Examination (MMSE) Score were 83.47 ± 6.45 years and 25.61 ± 6.45 points, respectively. All participants were comprehensively assessed for executive function, learning and delayed memory, language, processing speed and visuoperceptual skills. Compared to the AD group, the patients with DD in this study exhibited significantly worse visuoperceptual ability, but significantly better memory skills. These findings suggested that late‐onset DD shared common profiles of cognitive deficits with DLB rather than with AD; however, there were limited longitudinal follow‐up data.

### 2.3 A Follow‐Up of DD in Late Life

In later life, newly emergent delusions often arise within the complex interplay of biological and psychosocial factors, medical comorbidities, age‐related brain changes and social isolation. Cross‐sectional diagnostic criteria are poorly suited for disentangling these influences or capturing evolving neurodegenerative processes. Therefore, longitudinal follow‐up studies of patients with late‐onset DD are required to clarify the proportion of patients who subsequently develop neurodegenerative dementia. In a study by Nagendra and Snowdon [26], among 51 patients followed for a mean of 36.6 months, four subsequently developed dementia and two developed MCI. In contrast, a large cohort study of patients with VLOSLP reported a median interval of 1.9 years from VLOSLP diagnosis to dementia diagnosis [5]. However, VLOSLP encompasses heterogeneous psychotic disorders, including DD, which limits direct comparison between these studies. The strict inclusion criteria for DD applied by Nagendra and Snowdon [26] might be associated with the finding that the majority of patients did not progress to dementia over the short term. Given the frequent refusal of medication and psychiatric care among patients with DD, the true long‐term rate of conversion to dementia remains unclear. Although a clinically meaningful subset may represent early manifestations of neurodegenerative diseases, identifying this subgroup remains unresolved.

### 2.4 Pre‐Psychosis in MBI

The MBI conceptualises the later‐life emergence of persistent neuropsychiatric symptoms as manifestations of prodromal neurodegenerative disease processes rather than purely psychological reactions [29]. The MBI comprises 5 domains: decreased motivation (apathy), affective dysregulation (mood/anxiety symptoms), impulse dyscontrol, social inappropriateness and abnormal perception or thought content (psychotic symptoms). Within this framework, through the construct of pre‐psychosis, delusions often present as mild suspiciousness or overvalued ideas rather than fixed, systematised beliefs. Hallucinations may occur, but are generally less frequent at this early stage and the presence of hallucinations alongside delusions may reflect a more advanced or clinically significant manifestation. The International Psychogeriatrics Association Psychosis in Neurodegenerative Disease Working Group emphasised that pre‐psychosis differs from VLOSLP in severity, phenomenology and trajectory, representing a subtle neuropsychiatric risk state rather than a primary psychotic disorder [30]. Consequently, despite the presence of predominant delusions, pre‐psychosis does not constitute a formal diagnosis of DD.

Longitudinal studies have demonstrated that persistent late‐life psychotic‐like symptoms are associated with an increased risk for subsequent cognitive decline and dementia, suggesting that these symptoms may represent early manifestations of neurodegenerative processes. In a population‐based cohort study involving 5742 participants with a mean age of 62 years (standard deviation, 6.91 years; range, 50–88 years), the most common Mild Behavioural Impairment Checklist (MBI‐C) items related to affective dysregulation were present in 34% and 38% of participants, according to self‐report and informant report, respectively [31]. In contrast, the least common items were those related to abnormal thoughts and perception (psychotic symptoms), which were present in only 3% and 6%. Notably, because this cohort did not undergo objective cognitive testing, the results may have been influenced by the inclusion of individuals with undiagnosed or very early‐stage MCI. This confounding factor was addressed in another large community‐based study, which strictly excluded individuals with MCI through comprehensive objective cognitive assessments. This study followed 12 452 cognitively intact older adults with a median age of 72 (interquartile range, 67–79; range, 60–104) for a median of 4.7 years [32]. Reflecting the strict exclusion of MCI, baseline psychotic symptoms were even rarer in this cohort, appearing in only approximately 1%–2% of participants depending on operational definitions. Despite their low prevalence, these symptoms confer the highest risk for subsequent dementia among all neuropsychiatric domains. Individuals exhibiting delusions or hallucinations demonstrated a markedly elevated dementia conversion rate compared to those without (18.7% vs. 5.7%), with a multivariable‐adjusted hazard ratio (HR) of approximately 3.6—substantially exceeding the risk associated with affective or agitation‐related symptoms (HR 1.5–1.6). Regarding dementia subtypes, psychotic symptoms carried a disproportionately higher risk for DLB (HR 13.9) than for AD (HR 2.2) or vascular dementia (HR 5.7). Given that participants meeting the criteria for primary psychotic disorders were excluded at baseline, the psychotic phenomena captured in this cohort likely represent attenuated or subthreshold manifestations corresponding to the concept of pre‐psychosis. Among participants without neuropsychiatric symptoms, one‐quarter developed dementia after a median interval of 12.0 years. This latency shortened to 10.1 years in the presence of affective symptoms, 9.1 years in the presence of agitation symptoms and plummeted to 4.1 years in the presence of psychotic symptoms. These differing intervals between the emergence of specific neuropsychiatric symptoms and the onset of dementia suggest that psychotic symptoms arise with a substantially shorter latency to overt cognitive decline compared to other behavioural symptoms. Furthermore, considering that the median baseline age of the participants who subsequently developed dementia was 79 years, psychotic symptoms as a manifestation of pre‐psychosis were presumed to emerge at a relatively advanced age.

## 3 Delusional Disorder as Psychiatric‐Onset Prodromal LBD

### 3.1 Terminology of LBD

As a clinical term, LBD remains incompletely defined [9, 33, 34]. Longitudinal follow‐up studies have demonstrated that most patients with isolated RBD eventually develop DLB or PD and, in rare cases, multiple system atrophy. Accordingly, isolated RBD has been proposed as a clinical phenotype within the spectrum of LBD [35]. A subset of patients diagnosed with pure autonomic failure (PAF) also develops DLB or PD [36]. Given the broad spectrum of clinical phenotypes preceding the development of DLB or PD, the term *prodromal LBD* has been proposed as a generic term before the fulfilment of the diagnostic criteria. In this article, *prodromal LBD* is clinically used to describe cases in which a clinical diagnosis of MCI cannot be made owing to overt psychiatric manifestations [10]. Given the lack of a precise definition of LBD beyond its use as a generic umbrella term, we used LBD as a pathological term to avoid conceptual ambiguity.

Pathologically, LBD is characterised by widespread Lewy body (LB) pathology, consisting mainly of phosphorylated α‐synuclein in both the central and peripheral nervous systems [9, 37, 38, 39]. Haematoxylin and eosin staining reveals that brainstem‐type LBs exhibit an eosinophilic core surrounded by a pale halo, whereas cortical‐type LBs appear as less well‐defined eosinophilic intraneuronal inclusions without a halo (Figure 1). These inclusions are more clearly visualised using α‐synuclein immunostaining. With regard to the distribution and severity of LB pathology, three pathological subtypes have been originally defined: brainstem‐predominant LBD (BLBD), transitional (limbic) LBD (TLBD) and diffuse neocortical LBD (DLBD) [34, 40, 41]. According to the pathological criteria for DLB, clinical presentation is typically associated with TLBD or DLBD [38]. Thus, cortical involvement in LB pathology is presumed to be essential for the development of full DLB syndrome. In contrast, BLBD largely corresponds to the pathological substrate of PD, when the marked neuronal loss in the substantia nigra (SN) is observed [41, 42]. To date, autopsy cases of isolated RBD largely had either BLBD (*n* = 3) or TLBD (*n* = 2) [43, 44, 45, 46]. A detailed pathological investigation of the SN demonstrated relative preservation of neuronal populations in this region, at approximately 40% of control levels, which is consistent with the absence of parkinsonism [45]. One case of TLBD accompanied by MCI further supports the concept that cognitive impairment reflects cortical involvement in LB pathology [9, 46]. Taken together, these observations suggest that the pathological substrate of prodromal DLB may correspond to early TLBD with the relative preservation of neuronal populations in the SN. According to the pathological criteria for DLB, the degree of neurodegeneration in the SN was semi‐quantitatively assessed (Figure 2) and documentation of the presence or absence of spontaneous parkinsonism during the lifetime was recommended [9].

**FIGURE 1:**
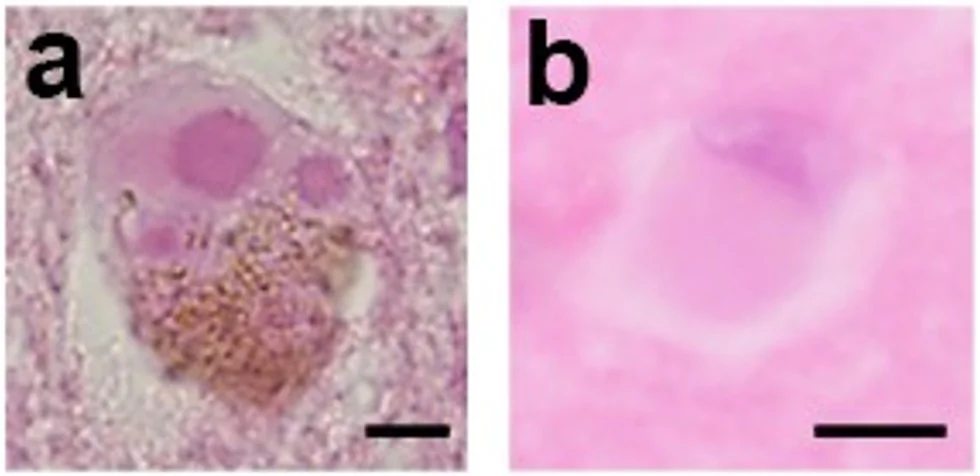
Microscopic findings of Lewy bodies. Lewy bodies (LBs) are eosinophilic intraneuronal inclusions stained with haematoxylin and eosin. The brainstem‐type LB has a pale‐staining halo (a), but the cortical type lacks the halo (b). Scale bar: 5 μm.

**FIGURE 2:**
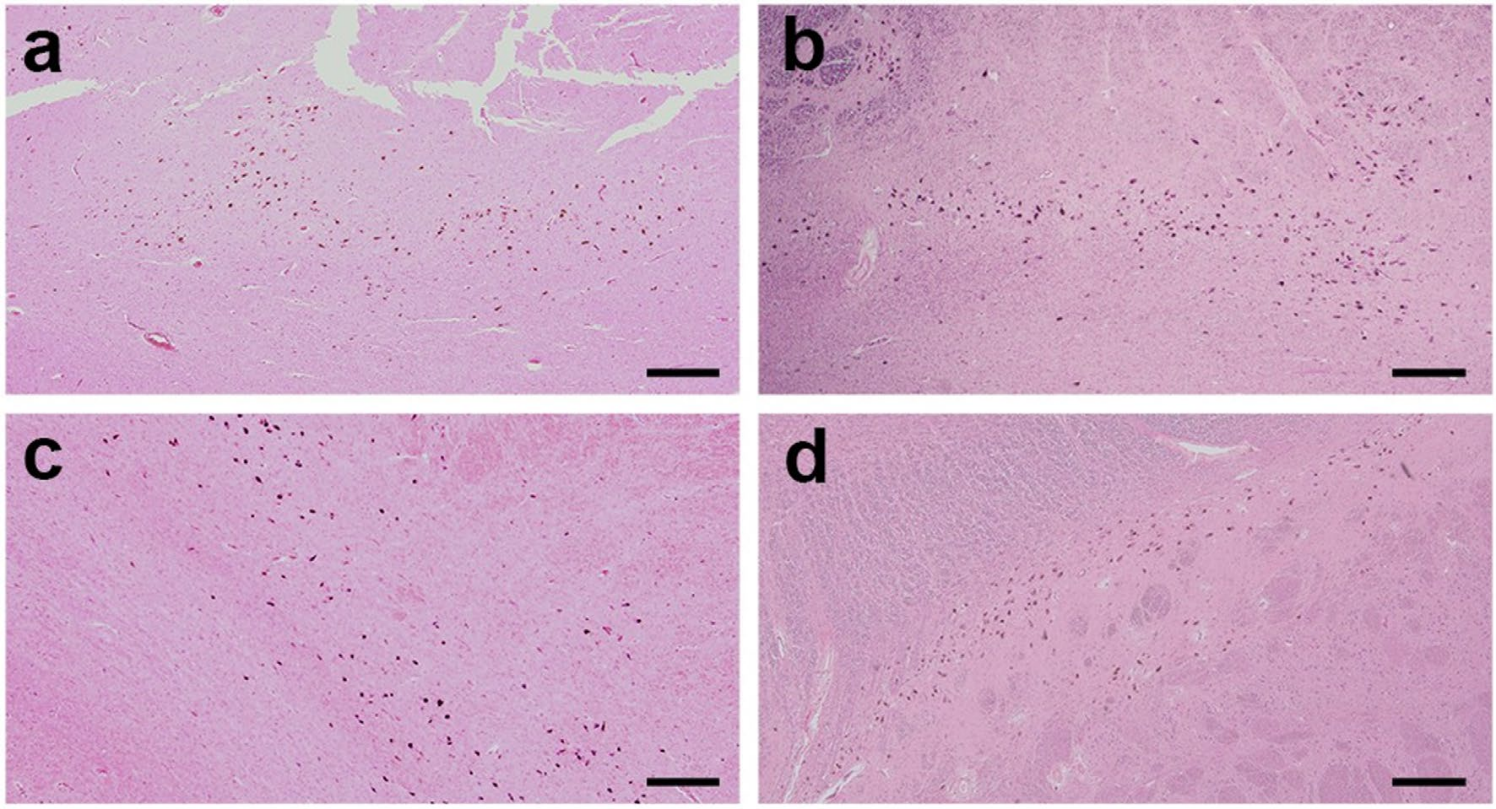
Nigral neurodegeneration in four patients with late‐onset delusional disorder as initial manifestation. Neuronal number in the substantia nigra on haematoxylin–eosin staining was semi‐quantitatively classified into mild loss in autopsy Case 1 (a) and autopsy Case 3 (c), none in autopsy Case 2 (b), and moderate loss in autopsy Case 4 (d). Scale bars: 200 μm.

### 3.2 A Review of Clinical Characteristics in Our Cases

We revisited our previously reported cases in which delusions were the initial manifestation of prodromal LBD [20, 21, 22]. Based on the clinical information reported in the original publications, one clinical case and four autopsied cases were considered to have late‐onset DD when their entire clinical courses were taken into account. Written informed consent had been obtained from their family for publication in connection with the original reports. The clinical profiles and courses are shown in Table 1 and Figure 3, respectively. No sex predominance was observed in this series. Two autopsy cases had family histories of psychiatric disorders. Along with living alone, events such as the death of a family member or relocation were considered psychosocial factors contributing to the occurrence of delusions. It is likely that somatic events such as legal blindness and hepatic encephalopathy influence the onset of delusions.

**TABLE 1:** Clinical profiles of patients with prodromal Lewy body disease manifesting as delusional disorder.

		Clinical Case 1 [18]	Autopsy Case 1 [19]	Autopsy Case 2 [20]	Autopsy Case 3 [19]	Autopsy Case 4 [19]
Sex		Male	Male	Male	Female	Female
Psychiatric family history		None	Brother (schizophrenia)	None	Sister (commit suicide)	Unknown
Medical comorbidities		Pneumoconiosis, atrial fibrillation, spinal spondylosis	Diabetes mellitus, chronic renal failure, blindness	Covert hepatic encephalopathy	Hearing loss	Controlled hyperthyroidism
Age at onset of delusions		67	56	66	60	54
Contents of delusions		Persecutory (pursuit, poisoning) delusion	Persecutory (poisoning) delusion	Persecutory (poisoning) delusion	Persecutory delusion	Delusion of poverty
Psychological events		Death of family	None	None	Relocation	None
Somatic events		None	Legal blindness	Covert hepatic encephalopathy	None	None
Onset age	Cognitive decline	76	68	None	85	73
	Dementia	76	None	None	85	76
	REM sleep behaviour disorder	None	None	None	None	None
	Parkinsonism	73 (Drug induced)	65 (Drug induced)	None	86 (Drug induced)	76
	Visual Hallucination	None	None	66^a^ (Transient)	88	73
	Cognitive fluctuation	None	None	66^a^	92	85
Age of death		—	69	66	93	94
Hypersensitivity of antipsychotics		Present	Present	None	Present	None
Choline esterase inhibitor		Effective	No medication	No medication	No medication	No medication
Refusal of psychiatric care		Present	Present	Present	Present	Present

^a^ During overt hepatic encephalopathy in the last month of life, autopsy Case 2 exhibited non‐recurrent visual hallucinations and cognitive fluctuations, which were considered more likely attributable to hepatic encephalopathy than Lewy body disease based on their clinical presentation and temporal association.

**FIGURE 3:**
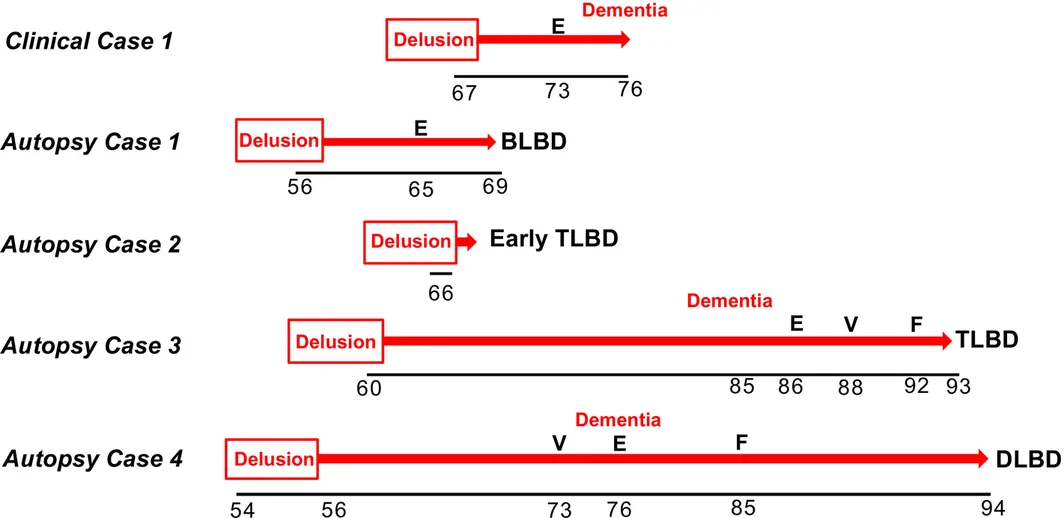
Clinical courses of patients with prodromal Lewy body disease manifesting as delusional disorder. Evolution of core clinical features of dementia with Lewy bodies based on the age in five cases. Age at death and pathological subtype of Lewy body disease at autopsy are also shown in four autopsied cases. Clinical Case 1: delusion—67; extrapyramidal symptoms (E)—73; and dementia—76. Autopsy Case 1: delusion—56; extrapyramidal symptoms (E)—65; and death—69. Autopsy showed BLBD. Autopsy Case 2: delusion—66; and death—66. Autopsy showed early TLBD. Transient, non‐recurrent visual hallucinations and cognitive fluctuations during overt hepatic encephalopathy in the last month of life were considered more likely attributable to hepatic encephalopathy than Lewy body disease, given their clinical presentation and temporal association. Autopsy Case 3: Delusion—60; dementia—85; extrapyramidal symptoms (E)—86; visual hallucinations (V)—88; fluctuating cognition (F)—92; and death—93. Autopsy showed TLBD. Autopsy Case 4: Delusion—54; visual hallucinations (V)—73; extrapyramidal symptoms (E)—76; dementia—76; fluctuating cognition (F)—85; and death—94. Autopsy showed DLBD. BLBD, brainstem‐predominant Lewy body disease; DLBD, diffuse neocortical Lewy body disease; TLBD, transitional Lewy body disease.

In clinical Case 1, delusions emerged at 67 years [20]. Administration of the antipsychotic brexpiprazole induced extrapyramidal symptoms, such as postural instability and falls. After switching to aripiprazole, the extrapyramidal symptoms were relieved without exacerbating agitation; however, the patient's delusions continued. After a clinical diagnosis of probable DLB was made, donepezil treatment was effective against the delusions. Considering that the administration of antipsychotics for delusions often causes drug‐induced parkinsonism, it is necessary to determine the optimal timing for the use of cholinesterase inhibitors.

In other autopsied Cases 1–4 [21, 22], the age at onset of delusions ranged from 54 to 67 years, with the delusions being predominantly persecutory. In our cases, three patients developed dementia in conjunction with core features within the typical age range of DLB onset. The durations from the onset of delusions to the development of DLB were 9, 22 and 25 years, suggesting a long‐term period of isolated delusions. In all cases reviewed, refusal of medication and psychiatric care was observed. Therefore, compulsory hospitalisation for treatment was required for all cases. Moreover, head computed tomography (CT) scans in autopsy Case 3 showed preserved medial temporal lobes, highlighting the difficulty in identifying psychiatric‐onset prodromal LBD based solely on morphological imaging [47].

In summary, the typical longitudinal course involved an onset in the 50s and 60s, with symptoms confined to persistent delusions for many years, followed by the development of dementia accompanied by core clinical features after approximately 75 years of age. In particular, the emergence of persistent visual hallucinations and parkinsonism was associated with the subsequent conversion to DLB. Notably, the initial delusional episodes were commonly triggered by both psychosocial and medical factors.

### 3.3 Neuropathological Substrate of Delusions in Patients With Prodromal LBD

To date, we have reported four patients presenting with DD as the initial manifestation of autopsy‐confirmed LBD [21, 22]. Their neuropathological findings are summarised in Table 2. Although clinicopathological correlations are often limited by the interval between symptom onset and autopsy, our cases had a wide range of pathological distributions, with various intervals between them (ranging from 3 months to 40 years). The two autopsied patients without dementia (autopsy Cases 1 and 2) had BLBD and early TLBD, respectively. In addition to BLBD, the minimal cortical involvement of LBs in autopsy Case 2 suggested a marginal type of DLB. Considering cortical involvement of LBs as a pathological substrate of global cognitive decline, the BLBD or early TLBD subtypes were consistent with the absence of overt dementia. In contrast, the other cases (autopsy Cases 3 and 4) had TLBD and DLBD, supporting a clinicopathological correlation with LBD in terms of developing dementia. Regarding nigral degeneration, the degree of neuronal loss in the SN was assessed according to the fourth CDLB neuropathological criterion [9, 48]. The minimum nigra neuronal loss in autopsy Cases 1 and 2 was consistent with the absence of spontaneous parkinsonism during their lifetimes (Figure 2a,b). In autopsy Cases 3 and 4, the relative preservation of neuronal populations in the SN may account for the absence of parkinsonism during the phase dominated by isolated delusions (Figure 2c,d). According to the ABC score of the National Institute on Ageing–Alzheimer's Association (NIA–AA) [49], autopsy Cases 1 and 2 showed scarce AD‐type pathology. In contrast, autopsy Cases 3 and 4 were assigned to the intermediate‐likelihood category of AD neuropathological changes. In terms of the DLB likelihood scheme, autopsy Cases 3 and 4 were assigned to the intermediate‐ or high‐likelihood category of DLB [9]. The observation of full DLB syndrome in autopsy Cases 3 and 4 supports the previous finding that groups with intermediate‐ or high‐likelihood categories of DLB pathology represent neuropathological substrates of DLB [21, 50, 51]. Autopsy Case 2 had a high likelihood of developing DLB; transient visual hallucinations and cognitive fluctuations were observed during overt hepatic encephalopathy in the last month of life, making their attribution to LBD uncertain. In addition to the marginal type of DLB, an interval of only 3 months until death may have limited clinical manifestations of DLB [22]. Regarding the neuropathological substrates of incident delusions, Mizutani et al. [52] reported that the accumulation of LBs in the dorsal raphe nucleus of the brainstem is associated with delusions in LBD. In the earlier stage of the disease, LB pathology is often confined to the brainstem regions with minimum or absent cortical involvement. Importantly, such limited Lewy pathology may precede substantial neocortical involvement and nigral neuronal loss by many years. These observations suggest that persistent delusions can emerge in the context of relatively limited Lewy pathology before widespread cortical involvement and significant nigral degeneration become apparent. Together with the preservation of the medial temporal lobes, the relative preservation of nigral neuronal populations may also contribute to the difficulty in identifying DD associated with LBD using neuroimaging findings alone [47].

**TABLE 2:** Pathological findings in four autopsied cases with delusional disorder.

Lewy‐related pathology type			Autopsy Case 1	Autopsy Case 2	Autopsy Case 3	Autopsy Case 4
			Brainstem‐predominant	Limbic (Transitional)	Limbic (Transitional)	Diffuse neocortical
The severity of Lewy‐related pathology	Brainstem regions	IX‐X	2	2	3	2
		LC	2	2	3	2
		SN	1	0–1	2	2
	Basal forebrain/limbic regions	NBM	1	2	3	2
		Amygdala	2	2	3	4
		Transentorhinal	1	0–1	2	3
		Cingulate	0	0–1	1	2
	Neocortical regions	Temporal	0	0–1	1	2
		Frontal	0	0–1	1	NE
		Parietal	NE	0	1	NE
Brain weight, g			1246	Not provided	1044	1100
The degree of neuronal loss in the SN			Mild	None	Mild	Moderate
Braak neurofibrillary tangle staging			1	1	4	4
Thal phase			0	1	4	4
CERAD neuritic plaque score			None	None	Sparce	Frequent
AD neuropathologic change (NIA‐AA)			Not	Low	Intermediate	Intermediate
Likelihood of DLB pathology			Low	High	Intermediate	High
Saito Argyrophilic grain stage			0	0	0	0
Large infarct			0	0	0	0
Cerebral amyloid angiopathy score			0	0	1	0
Arteriolosclerosis score			0	0	1	0

Abbreviations: AD, Alzheimer's disease; CERAD, Consortium to Establish a Registry for Alzheimer's Disease; DLB, dementia with Lewy bodies; IX, 9th cranial nerve nucleus; LC, locus coeruleus; NBM, nucleus basalis of Meynert; NE, not examined; NIA‐AA, National Institute on Ageing in collaboration with the Alzheimer's Association; SN, substantia nigra; X, 10th cranial nerve nucleus.

## 4 Long‐Term Nature of Prodromal LBD

### 4.1 Prodromal LBD as a Prolonged and Heterogeneous Disorder

DLB is characterised by progressive cognitive impairment accompanied by motor symptoms, sleep–wake disturbances, dysautonomia and psychiatric symptoms. Recent DLB management guidelines recommend a comprehensive assessment of these five domains to guide therapeutic strategies [53]. The severity and presentation of these symptoms vary widely among individuals in cross‐sectional assessments; they increasingly overlap as the disease progresses, ultimately resulting in a convergent clinical presentation of the full DLB syndrome.

Pathologically, DLB is rooted in LBD, defined by the abnormal aggregation of α‐synuclein in the form of LBs and Lewy neurites. Rather than representing a single clinicopathological entity at the onset, LBD encompasses a broad spectrum of clinical phenotypes, including PD, isolated RBD and PAF. These phenotypes share LB pathology but are presumed to differ in the severity and anatomical distribution of the underlying pathology at their earliest clinical manifestations. They typically manifest in the 50s to 60s. When the clinical manifestations are initially restricted to motor, sleep or autonomic dysfunction, the phenotype may remain relatively stable for a prolonged period, with subsequent progression to DLB typically reported from around age 75 onward [21]. In addition to these clinical phenotypes, the clinical trajectory of psychiatric‐onset prodromal LBD toward evolving DLB is illustrated schematically in Figure 4. This trajectory represents a hypothesis‐generating clinicopathological model rather than an established natural history of progression to DLB. The clinical trajectories of each phenotype are discussed in detail below.

**FIGURE 4:**
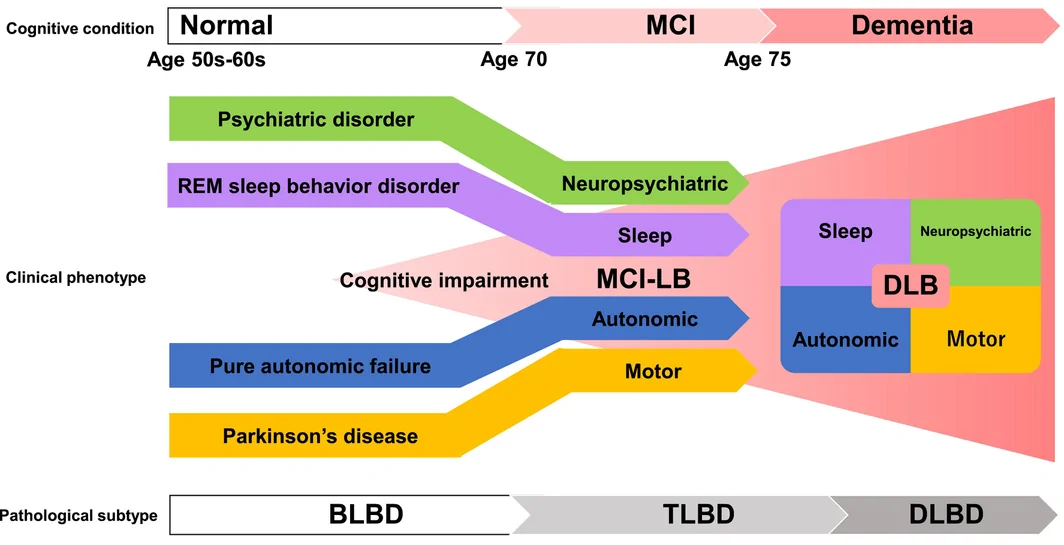
Conceptual diagram illustrating a hypothetical clinical trajectory toward evolving dementia with Lewy bodies. Dementia with Lewy bodies (DLB) is characterised by progressive cognitive impairment accompanied by four clinical domains: motor symptoms, dysautonomia, sleep–wake disturbances and psychiatric symptoms. When the initial clinical features manifest as an isolated condition within a single domain in the late 50s to 60s, the clinical course can present as Parkinson's disease, pure autonomic failure, isolated REM sleep behaviour disorder or psychiatric disorder. Clinical presentations of psychiatric disorders include delusional disorder and depressive disorder. As cognitive decline emerges—typified by mild cognitive impairment with Lewy bodies (MCI‐LB) as the most common clinical phenotype of prodromal DLB—these individual symptom domains progress to varying degrees of severity over time, ultimately culminating in overt dementia with the full‐blown DLB syndrome around the mid‐70s and later. BLBD, brainstem‐predominant Lewy body disease; DLB, dementia with Lewy bodies; DLBD, diffuse neocortical Lewy body disease; MCI, mild cognitive impairment; MCI‐LB, mild cognitive impairment with Lewy bodies; TLBD, transitional Lewy body disease.

#### 4.1.1 PD

A clinicopathological study involving 111 patients fulfilling the diagnostic clinical criteria for PD was conducted using the Queen Square Brain Bank in the United Kingdom [54]. Based on the clinical subtype classification of the Parkinson's Progression Markers Initiative (PPMI) [55], cases were categorised into 3 groups: mild‐motor‐predominant (*n* = 54 [48.7%]); diffuse‐malignant (*n* = 18 [16.2%]); and an intermediate group that did not fulfil the criteria for either subtype (*n* = 39 [35.1%]).

According to the PPMI classification, low cerebrospinal fluid amyloid‐beta levels, concomitant cognitive impairment, autonomic dysfunction, RBD and rapid disease progression are the characteristic features of the diffuse‐malignant subtype, which overlaps with the clinical phenotype of DLB [54, 55]. In the Queen Square cohort, the mild‐motor‐predominant group had a younger mean age at diagnosis (58.2 years), a longer mean interval from diagnosis to dementia onset (17.1 years) and a longer disease duration (20.2 years) [54]. In contrast, the diffuse malignant group had an older mean age at diagnosis (70.3 years), poorer responsiveness to L‐dopa, a shorter mean period from diagnosis to dementia onset (4.1 years) and a shorter mean disease duration (8.1 years). Although the clinical course differed between the two groups, the mean age at death was similar (78.3 vs. 78.4 years) and no significant differences were observed in the autopsy findings across the neuropathological rating scales. These findings suggest that when PD begins in the late 50s with isolated motor symptoms and without overt non‐motor manifestations, the motor symptoms progress slowly in isolation, whereas late‐onset PD tends to exhibit clinical features resembling those of DLB. In both groups, dementia developed around 75 years of age and the clinical phenotypes and pathological findings at death were similar between them.

#### 4.1.2 Isolated RBD

The International RBD Study Group demonstrated that isolated RBD is a multisystem prodromal synucleinopathy rather than a disorder confined to abnormal behaviour during REM sleep [56]. At study entry (1280 patients with mean age of 66.3 years), abnormalities across multiple domains—including motor, cognitive and autonomic function—were common and many independently predicted phenoconversion. The strongest predictors were subtle motor and mild cognitive impairments, while constipation and erectile dysfunction increased the risk of conversion. Given a median time to phenoconversion of 8.0 years, overt DLB or PD would be expected to emerge in many patients at around their mid‐70s. In a subsequent multicentre cohort study focusing on DLB, participants were enrolled at a mean age of 72.5 years and developed DLB after a mean follow‐up of 2.8 years, corresponding to a mean age of approximately 75 years [57]. Longitudinal cognitive trajectories have demonstrated that cognitive decline is detectable for up to 10 years before phenoconversion, indicating that cognitive impairment already emerges during the isolated RBD stage in patients who subsequently develop DLB. However, a subset of patients remains stable for more than a decade, presenting solely with RBD symptoms without phenotypic conversion. Iranzo et al. [58] followed 20 individuals who did not develop DLB or PD during a mean follow‐up period of 12 years after the diagnosis of isolated RBD. To determine whether these patients represented prodromal LBD, the authors investigated the presence of LB‐related clinical features. Although modest, these features were significantly more prevalent among the patients than in the controls. Thus, even in the absence of overt clinical progression, the findings strongly suggested the presence of underlying LBD. In their cohort, the mean age at the onset of isolated RBD symptoms was 54.9 years and the mean age at diagnosis was 60.6 years.

#### 4.1.3 PAF

In a prospective longitudinal cohort study in the United States, the mean age at onset of neurogenic orthostatic hypotension was 63 ± 14 years [59]. Notably, a younger age at onset was associated with a longer period during which clinical manifestations remained restricted to isolated autonomic dysfunction. Among the patients who developed PAF at or before 60 years of age, the mean duration of this isolated autonomic phase before progression to DLB or PD was 18.1 years, whereas among those with an onset after 60 years of age, the corresponding duration was significantly shorter at 8.4 years. Focus on the 13 patients who ultimately progressed to DLB reveals that the mean age at the onset of PAF was 66 ± 7 years and the mean duration of the PAF phase was 10 ± 5 years, indicating that DLB typically manifested when patients reached their mid‐70s. Within this cohort, the coexistence of probable RBD was strongly associated with progression to DLB. Furthermore, olfactory dysfunction and subtle motor signs at baseline served as key predictive factors for the subsequent development of DLB. These findings demonstrate that clinical features extending beyond dysautonomia are highly valuable for predicting phenoconversion to DLB.

#### 4.1.4 Psychiatric‐Onset Prodromal LBD

In a clinicopathological study of 165 consecutive autopsy cases from the Brain Bank of the Department of Psychiatry at Nagoya University, 20 patients with LBD who underwent autopsies in psychiatric hospitals were identified [21]. Based on current classifications of prodromal DLB, formal clinical criteria of MCI‐LB were established. However, psychiatric symptoms or delirium are not uncommon during the predementia phase in patients with MCI‐LB, suggesting blurred boundaries between these classifications [60, 61]. Therefore, cases in which both clinical features emerged alongside progressive cognitive decline were classified as the MCI‐onset subtype. In contrast, psychiatric‐ or delirium‐onset subtypes were rigorously defined only when each episode occurred in isolation, with no detectable cognitive decline remaining after full recovery. Based on these definitions, prodromal LBD cases were classified into the following subtypes: MCI‐LB (*n* = 12 [60%]); psychiatric‐onset prodromal LBD/DLB (*n* = 4 [20%]); delirium‐onset DLB (*n* = 2 [10%]); and motor‐onset DLB (*n* = 2) [10%]. In the four psychiatric‐onset cases, anxiety and delusions emerged between 54 and 60 years of age and psychiatric symptoms alone persisted for a prolonged period. Three patients predominantly exhibited delusions without overt progressive cognitive decline, consistent with DD. Except for one patient who died at the age of 69 years, dementia developed between 76 and 85 years and was accompanied by core clinical features, fulfilling the diagnostic criteria for DLB. Across the three prodromal DLB subtypes and the motor‐onset group, the mean age at dementia onset ranged from 76 to 83 years, showing no significant differences among the groups. These findings indicate that even when psychiatric symptoms emerge in isolation between their 50s and 60s, the subsequent onset of dementia—accompanied by core clinical features—corresponds to the common age of onset for DLB. Clinicopathological analyses revealed no significant differences in age at death or neuropathological findings among the groups classified according to their initial symptoms, consistent with observations from the Queen Square PD Brain Bank [54]. Notably, coexisting psychiatric symptoms were identified before the development of overt dementia in 41.6% of the patients in the MCI‐onset group. Considering that these psychiatric symptoms emerged alongside cognitive decline rather than as isolated manifestations, these cases were better conceptualised as neurocognitive disorders rather than as primary psychiatric disorders according to the DSM‐5‐TR. In fact, none of the patients fulfilled the clinical criteria for DD. The median age at onset in the MCI‐onset group was 78.5 years, which is consistent with the demographics of MBI reported previously [29, 30, 31]. In contrast, all our cases required hospitalisation for psychiatric treatment, suggesting a difference in clinical severity compared with MBI.

Regarding depression and apathy, which are also supportive clinical features of DLB, late‐onset depressive disorder is the most common clinical phenotype in psychiatric‐onset prodromal DLB [10]. In our reported cases, many patients with first depressive episodes between their 50s and 60s exhibited long‐lasting, isolated depressive presentations [62, 63]. Specifically, two patients experienced their first depressive episodes at 53 and 59 years of age and remained diagnosed with major depressive disorder for the subsequent 30 and 21 years of their lives, respectively [62]. Both patients exhibited polysomnography‐confirmed RBD and abnormal findings on iodine‐123 metaiodobenzylguanidine (^123^I‐MIBG) myocardial scintigraphy. Considering the association between RBD and depression reported in previous studies [13, 64], the prolonged course of depressive disorder associated with LBD was therefore not unexpected. In contrast, our reported patients with symptom onset in their mid‐70s or later were more likely to convert to DLB within several years [65, 66, 67]. A recent systematic review of isolated RBD confirmed a high prevalence of depression, apathy and anxiety in patients with isolated RBD [64]. Regardless of the specific psychiatric presentation acting as a supportive feature, individuals initially presenting with primary psychiatric disorders between their 50s and 60s may develop DLB after an extended latency period. Because the number of our reported cases is limited, further studies are warranted to clarify the relationship between late‐onset psychiatric manifestations and the trajectory of evolving DLB.

## 5 Conclusion and Future Directions

DLB is fundamentally rooted in underlying LBD, encompassing a wide clinical spectrum that includes PD, isolated RBD, PAF and psychiatric manifestations. Although DLB is clinically managed through the comprehensive assessment of five distinct symptom domains—comprising cognitive impairment, motor symptoms, sleep–wake disturbances, dysautonomia and psychiatric manifestations—these presentations diverge significantly in their early stages. Phenotypes such as PD, isolated RBD and PAF typically manifest in the 50s and 60s. When the initial clinical features remain restricted to isolated motor, sleep, autonomic dysfunction or psychiatric manifestations, we hypothesise that the condition often follows a prolonged and stable course before converging into the full‐blown DLB syndrome typically around the mid‐70s and later. Across these diverse prodromal trajectories, individual symptom domains develop to varying degrees over time. While MCI‐onset DLB represents the most common clinical subtype, other trajectories begin with isolated non‐cognitive manifestations, yet their clinical manifestations may increasingly overlap and converge as the disease progresses.

According to our small series, late‐onset DD presenting as a prodromal LBD is clinically characterised by the onset of isolated delusions in the late 50s to 60s, a stable long‐term course without the development of core features and subsequent conversion to DLB around the mid‐70s and later. Given this trajectory, which differs from the common clinical course of DLB, distinguishing DD from broader categories of late‐onset psychosis may help refine future research. Neuropathologically, this prolonged psychotic phase likely reflects a relatively restricted distribution of Lewy pathology, wherein preserved nigral and neocortical integrity delays the manifestation of parkinsonism and global cognitive decline. Furthermore, our clinicopathological considerations suggest that conventional structural neuroimaging as well as functional modalities such as dopamine transporter imaging may fail to detect the abnormalities during this early isolated phase [47]. Therefore, incorporating molecular biomarkers, such as α‐synuclein seed amplification assays (e.g., RT‐QuIC), is expected to improve the accuracy of early in vivo diagnoses and may help reduce the risk of antipsychotic hypersensitivity [68, 69, 70]. A biologically defined approach to disease classification may facilitate the identification of prodromal LBD in patients with late‐onset psychiatric disorders [71, 72]. From the perspective of intervention for DLB, this strategy may provide a potential therapeutic window for future disease‐modifying therapies in terms of preventing the onset of dementia. Finally, accumulating well‐characterised clinical and pathological cases will be essential to further delineate the clinical trajectory and refine the diagnostic framework for prodromal LBD.

## Funding

This research was supported by the Japan Society for the Promotion of Science (JSPS) KAKENHI [Grant Number JP26K10487] and the Japan Agency for Medical Research and Development (AMED) [Grant Numbers JP21wm0425019 and JP25wm0625126].

## Ethics Statement

The authors have nothing to report.

## Conflicts of Interest

The authors declare no conflicts of interest.

## Data Availability

No new data were generated or analysed for this article. All clinical and pathological information discussed in this article was obtained from previously published reports cited in the manuscript.
